# Correlation of Near-Infrared Spectroscopy (NIRS) with Invasive Arterial Pressure Monitoring during Aortic Coarctation Surgery in Pediatric Patients

**DOI:** 10.3390/healthcare12181884

**Published:** 2024-09-20

**Authors:** Jelena Pjevalica Dragic, Tatjana Zecevic, Ivan Divac, Andrija Pavlovic, Dejan Bisenic, Luka Stanisic, Jasna Kalanj, Igor Stefanovic, Dejan Nikolic, Ivana Petrov, Vladimir Milovanovic

**Affiliations:** 1 Department of Cardiac Surgery, University Children’s Hospital, 11000 Belgrade, Serbia; zecevic@gmail.com (T.Z.); divac.ivan@yahoo.com (I.D.); dejanbisenic@gmail.com (D.B.); kalustanisic@gmail.com (L.S.); vmilovanovic1972@yahoo.com (V.M.); 2 Department of Cardiology, University Children’s Hospital, 11000 Belgrade, Serbia; andrijapavlovic88@gmail.com (A.P.); jessiee1973@gmail.com (J.K.); igorstefanovic@yahoo.com (I.S.); 3 Department of Physical Medicine and Rehabilitation, University Children’s Hospital, 11000 Belgrade, Serbia; denikol27@gmail.com; 4 Faculty of Medicine, University of Belgrade, 11000 Belgrade, Serbia; 5 Department of Anesthesiology and Intensive Care, University Children’s Hospital, 11000 Belgrade, Serbia; kikapetrov@hotmail.com

**Keywords:** near-infrared spectroscopy, aortic coarctation, pediatric patients, pressure gradient monitoring, invasive arterial pressure

## Abstract

Aortic coarctation surgery in pediatric patients requires the placement of two arterial cannulas to monitor pressure gradients and surgical correction adequacy. Near-infrared spectroscopy (NIRS) monitoring provides insight into regional blood flow. This study aimed to investigate the correlation between NIRS values and invasive arterial pressures, exploring NIRS monitoring as a potential substitute for arterial cannulation. In a cohort of 21 consecutive pediatric patients undergoing aortic coarctation surgery, recordings of NIRS and invasive arterial pressure values were evaluated at various time intervals. Pearson correlation evaluated the relationship between NIRS values and invasively measured arterial pressures. Moderate to strong correlations were observed between the mean arterial pressure (MAP) of the upper and lower arteries and cerebral (rSO_2_-C) and somatic (rSO_2_-S) NIRS values 5 min after cross-clamp placement (r = 0.621, *p* = 0.003; r = 0.757, *p* < 0.001). Strong correlations were found 15 min after cross-clamp placement (r = 0.828, *p* = 0.002; r = 0.783, *p* = 0.004). Before transfer to the ICU, a strong correlation existed between the upper artery MAP and rSO_2_-C (r = 0.730, *p* < 0.001), but there was no correlation between the lower artery MAP and rSO_2_-S. These findings are promising, but further studies are required to validate it as a reliable substitute for invasive pressure monitoring in this patient population.

## 1. Introduction

Aortic coarctation (CoA) accounts for up to 8% of all congenital heart diseases [1,2,3]. Surgical correction in pediatric patients typically occurs in early infancy, with more than 60% of repairs conducted before the age of 3 months [4].

During the induction of anesthesia for aortic coarctation surgery, the current standard of care implies the placement of two arterial cannulas to monitor the pressure gradient and assess the adequacy of surgical correction [1,5]. One cannula is positioned in the artery of the right arm (preductal position), while the other is typically inserted in the femoral artery (postductal position). Because the patients are usually neonates or small infants, the placement of both cannulas, although preferable, is not always feasible. Furthermore, although the overall major complication rate of arterial cannulation for monitoring purposes in children is low (0.2%) [6], the presence of congenital heart disease and newborn age are shown to be independently associated with increased thrombotic risk [7]. One of the hallmarks of aortic coarctation is the reduction in or the absence of femoral pulses [8], making it even more difficult to place the cannula in the femoral artery. To ensure accurate arterial pressure reading and pressure gradient monitoring, during the operation and afterward in the intensive care unit, placement of the cannula in the right arm artery and femoral artery is necessary. This necessity places significant responsibility on the attending anesthesiologist, making arterial cannulation mandatory part of intraoperative monitoring. Also, this sometimes significantly extends the duration of the induction of anesthesia, potentially worsening the patient’s preoperative state. While direct cannulation of the aorta can suffice for intraoperative monitoring in extreme situations, these patients lack the necessary monitoring during the postoperative recovery.

Near-infrared spectroscopy (NIRS) monitoring during cardiac surgery has gained favor in the last decade, especially in the pediatric population. Unlike pulse oximetry, NIRS-based cerebral and tissue oximeters do not include plethysmography, so there is no differentiation between arterial and venous blood. Consequently, cerebral and tissue oximeters do not provide an indicator of oxygen delivery but rather provide information on the balance between regional oxygen supply and demand [9]. This allows NIRS measurements to be obtained even in the absence of a pulse, such as during cardiopulmonary bypass (CPB) or during the cross-clamp time in aortic coarctation surgery. Because of the variability in arterial, venous, capillary, and nonvascular tissue ratios, baseline values vary between subjects by approximately 10%. This makes NIRS monitoring more suitable for use as a trend monitor rather than an absolute measure of tissue oxygenation [9,10,11,12]. Most clinical and research applications of NIRS monitoring in pediatric cardiac patients focus on cerebral (rSO_2_-C) oximetry values and the correlation between the values and patient outcome [9,13,14,15,16,17]. Berens et al. found that in pediatric patients undergoing aortic coarctation repair, monitoring of the somatic thoracodorsal region (rSO_2_-S) with a NIRS sensor provides real-time trend information on regional oxygenation below the aortic cross-clamp. The decline in rSO_2_-S during aortic cross-clamp was rapid and large in neonates and young infants, which suggested impairment of regional perfusion, likely attributed to insufficient collateral circulation [18].

This study aimed to determine whether a correlation exists between NIRS values and invasive arterial pressure values to assess if NIRS monitoring can serve as a substitute for invasive arterial pressure in situations where the placement of an arterial cannula, either in the right arm or femoral artery, is challenging or not feasible.

## 2. Materials and Methods

This is a retrospective study of pediatric patients who underwent isolated repair of aortic coarctation without the use of cardiopulmonary bypass.

### 2.1. Patients

**Figure 1:**
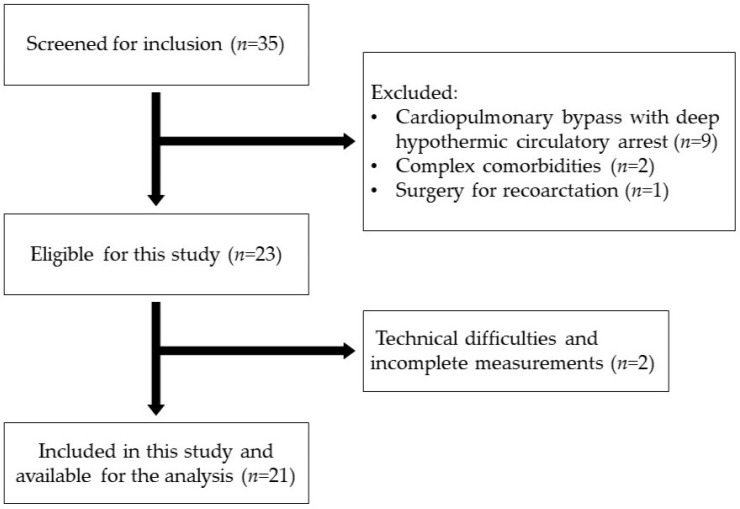
Study flowchart.

Between November 2022 and March 2024, a total of 35 pediatric patients underwent surgery for aortic coarctation at the University Children’s Hospital, Belgrade, Serbia. This study included patients who underwent isolated aortic coarctation repair without the use of cardiopulmonary bypass. Exclusion criteria were based upon the influence that it might have on hemodynamics and measurements of NIRS. The exclusion criteria were as follows: (1) use of cardiopulmonary bypass; (2) correction of complex congenital heart disease alongside aortic coarctation; (3) congenital conditions affecting NIRS reliability. Of the total number, nine patients were excluded due to the use of cardiopulmonary bypass and cardioplegia with deep hypothermic circulatory arrest. One patient with Taussig–Bing syndrome (transposition of the great arteries with a large ventricular septal defect) was excluded because the operation included an arterial switch and VSD closure. Another patient with Dandy–Walker syndrome was excluded due to central nervous system anomalies that compromised cerebral NIRS reliability. Two additional patients were excluded due to technical difficulties and the lack of serial NIRS measurements due to immediate postoperative transfer to another hospital. Figure 1 illustrates the study flowchart.

### 2.2. Invasive Arterial Pressure

Direct arterial cannulation was performed using 20-, 22-, or 24-gauge cannulas, depending on the patient’s age. The first cannula was placed in the right arm—upper artery (radial, brachial, or axillar artery), and the second cannula in the leg—lower artery (left or right femoral artery).

### 2.3. NIRS Monitoring

Cerebral and somatic oxygenation was monitored using an INVOS Cerebral Somatic Oximeter (Medtronic™) (Minneapolis, MN, USA) with neonatal or pediatric sensors. The cerebral sensor was positioned from the base of the nose to the left side of the forehead, measuring regional cerebral oxygenation (rSO_2_-C). The somatic sensor was positioned beneath the right rib cage, extending from the mid-axillary line to the back toward the spine. This sensor measured regional somatic oxygenation (rSO_2_-S) and provided insights into the saturation levels in the organs and tissues beneath the cross-clamp.

NIRS values recorded before the surgical incision (cerebral and somatic) served as a reference point against which all subsequent NIRS values were compared (cerebral for cerebral, somatic for somatic). A significantly low NIRS value was defined as either a decrease of 20% or more from the reference value or an absolute value of less than 45.

### 2.4. Data Collection

Invasive arterial pressure from both arteries and NIRS values from both sensors were recorded simultaneously, at various time intervals. Recordings were obtained from intraoperative patient records at 6 time points:

- Before the surgical incision (after anesthesia induction);
- Before the placement of the cross-clamp;
- 5 min after the placement of the cross-clamp;
- 15 min after the placement of the cross-clamp;
- 5 min after the removal of the cross-clamp;
- Before the transfer of the patient to the intensive care unit (ICU).

The average duration from cross-clamp removal to ICU transfer ranged from 30 min to 1 h.

### 2.5. Data Analysis

Statistical analysis was conducted using IBM SPSS Statistics (Version 27, IBM Corporation, Armonk, NY, USA). The normality of data was assessed using the Shapiro–Wilk test. Pearson correlation was used to evaluate the relationship between cerebral NIRS and the mean arterial pressure (MAP) of the upper artery, as well as between the somatic NIRS and the MAP of the lower artery.

## 3. Results

### 3.1. Patient Characteristics

Twenty-one patients met the inclusion criteria and were included in this study. The median patient age on the day of the operation was 43 days, ranging from 10 days to 10 years. This cohort comprised eight neonates, nine infants, and four children older than one year, with a male predominance (*n* = 15, 71%). The baseline patient characteristics are summarized in Table 1.

### 3.2. Anesthesia Management

Seventeen patients received thiopental for the induction of anesthesia, two patients received propofol, one patient received ketamine, and one patient was anesthetized only with sevoflurane. Anesthesia was maintained with sevoflurane, rocuronium, and fentanyl in all patients, in a standardized manner. There were no significant differences between NIRS measurements and different anesthetic groups (*p* ≥ 0.05).

### 3.3. Cannula Placement

The first cannula was placed in the right arm in all patients except one, for whom measurements were taken using the left radial artery. Twelve patients had the cannula in the right brachial artery, four patients in the right radial artery, and four patients in the right axillar artery. Eighteen patients had the second cannula in the right femoral artery, and three patients had it in the left femoral artery.

### 3.4. Surgery

In all patients, surgery was performed via left thoracotomy. Eighteen patients underwent extended end-to-end anastomosis (EETE), one patient underwent end-to-end anastomosis (8 years old), one patient had aortoplasty with a GORE-TEX patch (10 years old), and one patient had EETE with banding of the pulmonary artery. The mean cross-clamp time was 16.3 ± 3.8 min. There were no intraoperative complications such as bleeding or the need for revision of the anastomosis. There were no differences between NIRS measurements and different types of operations (*p* ≥ 0.05).

### 3.5. Intraoperative Measurements

Five minutes after the placement of the cross-clamp, 76% (16 patients) exhibited low rSO_2_-S, while 14% (3 patients) showed a drop in rSO_2_-C values.

Of the 11 patients with a cross-clamp time exceeding 15 min, 4 did not show a low rSO_2_-S. Among these patients, only 1 exhibited low rSO_2_-C, while the remaining 10 had normal rSO_2_-C values.

Five minutes after releasing the cross-clamp, 95% (20 patients) had normal rSO_2_-S values, and 81% (17 patients) had normal rSO_2_-C values. At the same time, 71% (15 patients) had an invasive systolic gradient <15 mmHg, while 95% (20 patients) had a MAP gradient <15 mmHg.

Before transfer to the ICU, 91% (19 patients) had normal rSO_2_-S values, 76% (16 patients) had normal rSO_2_-C, and 62% (13 patients) had an invasive systolic gradient <15 mmHg, with 1 patient exhibiting a MAP gradient ≥15 mmHg.

**Figure 2:**
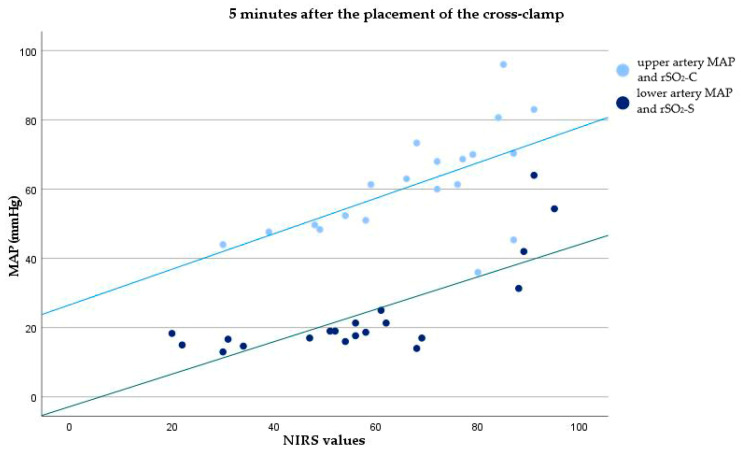
Correlation between cerebral NIRS values and MAP of the upper artery, and somatic NIRS values and MAP of the lower artery, 5 min after the placement of the cross-clamp. rSO_2_-C—regional cerebral oxygenation, rSO_2_-S—regional somatic oxygenation.

**Figure 3:**
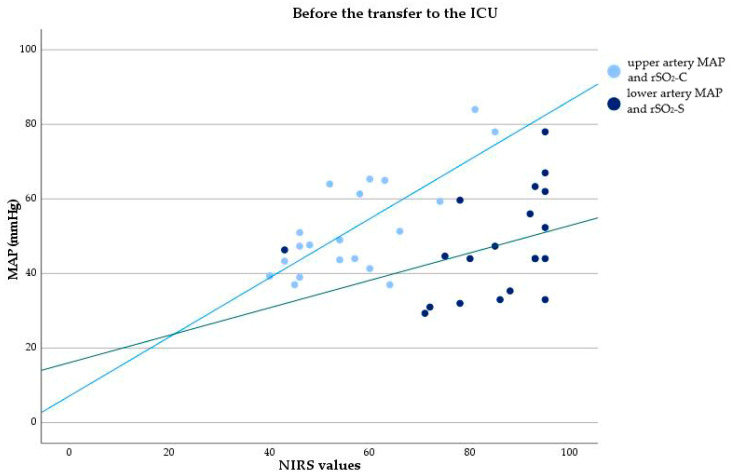
Correlation between cerebral NIRS values and MAP of the upper artery, and somatic NIRS values and MAP of the lower artery, before the transfer to the ICU. rSO_2_-C—regional cerebral oxygenation, rSO_2_-S—regional somatic oxygenation.

Moderate to strong correlations were observed between the MAP of the upper artery and lower artery and rSO_2_-C and rSO_2_-S 5 min after the placement of the cross-clamp, respectively (r = 0.621, *p* = 0.003; r = 0.757, *p* < 0.001). A strong correlation was found 15 min after the placement of the cross-clamp, respectively (r = 0.828, *p* = 0.002; r = 0.783, *p* = 0.004). Before transfer to the ICU, a strong correlation was evident between the MAP of the upper artery and rSO_2_-C (r = 0.730, *p* < 0.001), while there was no correlation between the MAP of the lower artery and rSO_2_-S. Table 2 displays all Pearson correlation coefficients and their corresponding *p*-values obtained in the operating room. Figure 2 and Figure 3 illustrate scatterplots depicting the correlation between rSO_2_-C and the MAP of the upper artery, as well as rSO_2_-S and the MAP of the lower artery at two time points: 5 min after the placement of the cross-clamp and before the transfer to the ICU.

## 4. Discussion

The main finding of our study is the moderate to strong correlation between the NIRS values and invasive arterial pressure measurements during aortic coarctation surgery. These results suggest that NIRS can provide a real-time, non-invasive monitoring of cerebral and somatic oxygenation, serving as a valuable tool for perfusion assessment and clinical decision making during surgery.

Our findings concur with the results of the study by Zhang et al. [19], which demonstrated a correlation between the somatic NIRS and lower-extremity MAP, as well as between the cerebral NIRS and upper extremity MAP during coarctation repair. However, their study was confounded by selective cerebral perfusion strategies, whereas our study provides a clearer view of these relationships in the absence of such contributing factors. To the best of our knowledge, no other studies have directly examined these correlations in a non-cardiopulmonary bypass setting, highlighting the broader use of NIRS monitoring in different surgical settings.

In our cohort, the expected drop in rSO_2_-S values following cross-clamp placement did not occur in five patients. Notably, four of these patients were older than 2 years. This observation aligns with the findings of Berens et al. [18], who reported that neonates and infants exhibit more significant drops in rSO_2_-S values due to inadequate collateral circulation, while older children show only minor changes, possibly due to more developed collateral circulation pathways. Farouk at al. [15] conducted a study on 11 consecutive patients, focusing on cerebral regional oxygenation during aortic coarctation repair. Although the age of their patients ranged from 8 days to 8 years (median 1.2 months), all of their patients showed a decrease in rSO_2_-S. In a randomized clinical trial, Moerman et al. studied how different blood pressure regulation strategies during aortic coarctation repair affected oxygen saturation in the brain, kidneys, and muscles, as measured by near-infrared spectroscopy. They also observed the effects of age on the maximal relative decrease in somatic NIRS values, which were explained by the lack of collateral circulation in younger patients [12]. In our study, the fifth patient who did not exhibit a drop in the rSO_2_-S value was a 2-month-old with a concomitant atrial septal defect. We assume that this patient did not exhibit low somatic NIRS values due to already developed collateral circulation, possibly because of the preductal position of the coarctation. Our results suggest that age and the presence of collateral circulation could affect somatic oxygenation during aortic cross-clamping.

Before transfer to the ICU, six patients had a low rSO_2_-C, while two patients had low rSO_2_-S values. In the study by Zhang et al. [19], aortic opening led to a remarkable increase in rSO_2_-S and a remarkable decrease in rSO_2_-C. This distinct change in the oxygen pattern is speculated to be due to hyperperfusion of the distal tissue beds and the simultaneous relative hypoperfusion (or a “steal” phenomenon) of the cerebral tissue. Although this study was conducted on patients who underwent surgery with cardiopulmonary bypass, we assume the same explanation can apply for the moment of releasing the cross-clamp in our study.

Additionally, before transfer to the ICU, eight patients had an invasive systolic gradient ≥15 mmHg, with one patient also showing a MAP gradient ≥15 mmHg. In our experience, patients are typically discharged after the initial surgery without suboptimal correction and residual gradients, despite the intraoperative revision of the anastomosis being rarely performed. Our data suggest that while invasive pressure gradients are currently the gold standard for assessing the adequacy of surgical correction, they may produce false positives for residual gradients immediately after surgery. The decision to re-evaluate anastomosis intraoperatively is often based on the subjective opinion of the surgeon. In contrast, NIRS offers a non-invasive, real-time assessment of tissue oxygenation that could better reflect the adequacy of surgical correction.

The results of our study demonstrated moderate to strong correlations between the MAP of the upper artery and the rSO_2_-C, and between the MAP of the lower artery and the rSO_2_-S, 5 min after the placement of the cross-clamp. Strong correlations were observed 15 min after cross-clamp placement. Prior to transfer to the ICU, a strong correlation persisted between the MAP of the upper artery and the rSO_2_-C, while the correlation between the MAP of the lower artery and the rSO_2_-S dissipated.

We hypothesize that rSO_2_-S values rapidly return to baseline values, or even exceed them, if the surgical correction is satisfactory. It is important to note that the lack of correlation with invasive arterial pressure at this time point may be due to the fact that invasive arterial pressure in the lower artery typically requires more time to return to expected values, whereas rSO_2_-S values may show immediate changes in response to surgical correction. Therefore, rSO_2_-S values could serve as an early indicator of surgical success or the need for reintervention, allowing the surgeon to make real-time decisions about re-evaluating the anastomosis based on the NIRS measurements during the same operation, rather than relying on invasive arterial pressure gradients and postponing the decision for the following days as in current practice. Further studies are necessary to investigate this hypothesis.

### Limitations

We acknowledge several limitations in our study. Firstly, it was a retrospective analysis conducted on a small patient cohort in a single-center setting. Exclusion criteria comprised patients undergoing surgery with cardiopulmonary bypass and deep hypothermic circulatory arrest. Future studies should consider including these patients to broaden the scope of investigation. Additionally, categorizing patients into age groups could enhance sample homogeneity. Furthermore, our study was limited by the absence of suboptimal surgical results in our patient cohort. Since we lacked cases of suboptimal surgical correction, we cannot draw conclusions about the correlation between NIRS values and invasive arterial pressure in these patients.

Additionally, our study is underpowered due to a small sample size (n = 21), as confirmed by power analysis. The retrospective nature and the rarity of coarctation of the aorta in the general pediatric population further limit the number of eligible patients, making it challenging to detect smaller, clinically relevant differences and increasing the risk of Type II errors. Despite these limitations, our findings offer valuable preliminary insights and suggest trends that warrant further investigation.

## 5. Conclusions

Our study reveals a moderate to strong correlation between both cerebral and somatic NIRS values and invasively measured arterial pressures in pediatric patients undergoing surgery for coarctation of the aorta. While these findings are promising, additional studies are required to validate NIRS monitoring as a reliable substitute for invasive arterial pressure monitoring in this patient population.

## Figures and Tables

**Table 1:** Baseline clinical characteristics of patients undergoing CoA repair.

Clinical and Demographic Characteristics	Overall (*n* = 21)
Median age at the intervention (days, range)	43 (10–3732)
Neonates (%)	38.0
Infants (%)	43.0
Children aged 1–18 yrs (%)	19.0
Age ≤3 months at the intervention (%)	76.0
Sex (male, %)	71.0
Body weight at the intervention (kg, SD)	7.56 ± 8.6
BSA at the intervention (kg/m^2^)	0.37 ± 0.28
PGE1 infusion (%)	32.0
Associated lesions	
Bicuspid aortic valve (%)	33.0
ASD (%)	24.0
VSD (%)	19.0
PAH (%)	9.0
Shone complex (%)	5.0
Hypoplastic aortic arch (%)	5.0
Intraoperative characteristics	
Lateral thoracotomy (%)	100.0
Cross-clamp time (min, SD)	16.33 ± 3.76
Extended end-to-end anastomosis (%)	86.0

**Table 2:** The Pearson correlation coefficients and corresponding *p*-values in the operating room.

Time Point	NIRS Sensor	Artery	Q	*p*-Value
Before the surgical incision	rSO_2_-C	upper artery MAP	0.632	0.002
	rSO_2_-S	lower artery MAP	0.482	0.027
Before the placement of the cross-clamp	rSO_2_-C	upper artery MAP	0.579	0.006
	rSO_2_-S	lower artery MAP	0.63	0.002
5 min after the placement of the cross-clamp	rSO_2_-C	upper artery MAP	0.621	0.003
	rSO_2_-S	lower artery MAP	0.757	<0.001
15 min after the placement of the cross-clamp	rSO_2_-C	upper artery MAP	0.828	0.002
	rSO_2_-S	lower artery MAP	0.783	0.004
5 min after the removal of the cross-clamp	rSO_2_-C	upper artery MAP	0.465	0.033
	rSO_2_-S	lower artery MAP	0.434	0.049
Before the transfer to the ICU *	rSO_2_-C	upper artery MAP	0.73	<0.001
	rSO_2_-S	lower artery MAP	0.353	0.127

* ICU—intensive care unit, rSO_2_-C—regional cerebral oxygenation, rSO_2_-S—regional somatic oxygenation.

## Data Availability

The raw data supporting the conclusions of this article will be made available by the authors upon request.
